# Proteomics Analysis of Normal and Senescent NG108-15 Cells: GRP78 Plays a Negative Role in Cisplatin-Induced Senescence in the NG108-15 Cell Line

**DOI:** 10.1371/journal.pone.0090114

**Published:** 2014-03-12

**Authors:** Wei Li, Wei Wang, Yan Li, Wenwen Wang, Tian Wang, Li Li, Zhiqiang Han, Shixuan Wang, Ding Ma, Hui Wang

**Affiliations:** 1 Cancer Biology Research Center, Tongji Hospital, Tongji Medical College, Huazhong University of Science and Technology, Wuhan, Hubei, P.R. China; 2 Department of Gynecology and Obstetrics, Nanfang Hospital, Southern Medical University, Guangzhou, P.R. China; University of Hong Kong, Hong Kong

## Abstract

Accelerated senescence (ACS) leading to proliferative arrest is a physiological mechanism of the DNA damage response that occurs during tumor therapy. Our experiment was designed to detect unknown genes that may play important roles in cisplatin-induced senescence and to illustrate the related senescence mechanism. Using 2-dimension electrophoresis (2-DE), we identified 5 protein spots with different expression levels in the normal and senescent NG108-15 cells. According to MALDI-TOF MS analysis, the 5 proteins were determined to be peptidylprolyl isomerase A (PPIA), peroxiredoxin 1 (PRX1), glutathione S-transferase mu 1 (GSTM1), vimentin (VIM) and glucose-regulated protein 78 (GRP78). Then, we investigated how cisplatin-induced senescence was mediated by GRP78 in the NG108-15 cells. Knockdown of GRP78 significantly increased P53 expression in NG108-15 cells. Additionally, 2-deoxy-D-glucose (2DG)-induced GRP78 overexpression protected the NG108-15 cells from cisplatin-induced senescence, which was accompanied by the obvious suppression of P53 and p-CDC2 expression. Inhibition of Ca^2+^ release from endoplasmic reticulum (ER) stores was also found to be associated with the anti-senescence effect of 2DG-induced GRP78 overexpression. In conclusion, we found 5 proteins that were differentially expressed in normal NG108-15 cells and senescent NG108-15 cells. GRP78 plays an important role in cisplatin-induced senescence in NG108-15 cells, mainly through its regulation of P53 expression and ER calcium efflux.

## Introduction

In normal cells, terminal proliferative arrest may result from terminal differentiation or replicative senescence. Treating normal cells with DNA-damaging drugs rapidly induces terminal proliferative arrest, which is accompanied by a senescent phenotype [1]. This phenotype includes morphological alterations, such as an enlarged and flattened shape with increased cytoplasmic granularity, the presence of polyploidy, and the expression of the pH-restricted, senescence-associated β-galactosidase (SA-β-gal) [2]–[3]. Nevertheless, unlike replicative senescence, this proliferation-arrested state is associated with rapid kinetics and telomere dysfunction without an overall net telomere shortening, which is referred to as accelerated senescence (ACS). In addition to normal cells, cultures of human cancer cells derived from solid tumors tend to undergo ACS following exposure to low doses of DNA-damaging drugs, such as cisplatin [4]. Furthermore, a recent study showed that the presence of SA-h-gal occurred in 41% of specimens from breast cancer patients who received induction chemotherapy but only in 10% of specimens from patients who underwent surgery without chemotherapy, which demonstrates that chemotherapy induces senescence *in vivo* [5]. These findings collectively suggest that ACS leading to proliferative arrest is a physiological mechanism of the DNA damage response that occurs during tumor therapy. However, the mechanisms of chemotherapy-induced senescence are still unclear.

In this study, we found that cisplatin could induce a senescence-like phenotype and growth arrest in NG108-15 cells. We then used 2-dimension electrophoresis (2-DE) to detect the key genes that may play important roles in cisplatin-induced senescence and to illustrate the related senescence mechanism. We found there were 5 differentially expressed proteins, peptidylprolyl isomerase A (PPIA), peroxiredoxin 1 (PRX1), glutathione S-transferase mu 1 (GSTM1), vimentin (VIM), and glucose-regulated protein 78 (GRP78), between the normal and senescent NG108-15 cells. GRP78, the most abundant and well-characterized glucose-regulated protein, is a major stress-induced chaperone that is localized to the endoplasmic reticulum (ER) [6]. GRP78 is thought to regulate the balance between cell survival and apoptosis [7]–[8]. Therefore, we further investigated the relationship between GRP78 and cisplatin-induced senescence in NG108-15 cells. It has been reported that ataxia telangiectasia mutated (ATM) pathway genes were also associated with chemotherapy-induced senescence [9]–[12]. Therefore, we also investigated the relationship between GRP78 and the ATM pathway in cisplatin-induced senescence.

## Materials and Methods

This study was reviewed and approved by the Ethics Committee of Tongji Hospital, Tongji Medical College, Huazhong University of Science and Technology (HUST). All experimental protocols were approved by the Institutional Animal Care and Use Committee of HUST, and the study was carried out in strict accordance with theARRIVE (Animal Research: Reporting of In Vivo Experiments) guidelines.

### Cell lines and agents

The mouse neuroblastoma and rat glioma hybrid NG108-15 cell line was purchased from the China Center for Type Culture Collection (Wuhan, China). Rabbit polyclonal antibodies against HP1-γ and GRP78 were purchased from PTG, Inc. (Wuhan, China). Rabbit polyclonal antibody against P53 was purchased from Boster Bio-Company (Wuhan, China). Mouse monoclonal antibody against P21 was purchased from Santa Cruz Biotechnology (CA, USA). Mouse monoclonal antibody against CDC2 was purchased from BD Biosciences (NJ, USA). Rabbit polyclonal antibody against (15-Tyr)-phosphorylated CDC2 was purchased from Cell Signaling Company (MA, USA). The goat anti-rabbit IgG labeled with FITC secondary antibody were purchased from Zhongshan Bio-Company (Beijing, China). The senescence-associated β-galactosidase staining kit was purchased from Cell Signaling Company (MA, USA). The CFSE fluorescent dye was purchased from DOJIN Company (Tokyo, Japan). Cisplatin was purchased from Sigma Company (NJ, USA). Caffeine was purchased from Alexion Company (Lausanne, Switzerland). Dulbecco's modified eagle medium (DMEM) was purchased from Gibco Company (MT, USA). 4′,6-diamidino-2-phenylindole (DAPI) was purchased from Sigma Company (NJ, USA). Fluo3-AM was purchased from Invitrogen Company (NY, USA). SA-β-Galactosidase Staining Kit was purchased from Cell Signaling company (MA, USA). 2-deoxy-D-glucose (2DG) was purchased from Sigma Company (NJ, USA). Agents for 2-DE were purchased from BioRad Company (CA, USA).

### Cell culture

NG108-15 cells were grown in DMEM supplemented with 10% fetal bovine serum in a humidified atmosphere of 5% CO_2_ and 95% air at 37°C. The cells were treated with cisplatin at the indicated concentrations when the cells were at a 30–40% cell density for 24 hours; the cells were then washed twice with PBS and maintained for 6 days in complete medium prior to harvesting. Caffeine was added 2 hours before cisplatin exposure. 2DG was added 24 hours before cisplatin exposure or transfection with small interfering RNAs.

### SA-β-galactosidase histochemical staining

Histochemical detection of SA-β-gal was performed with the SA-β-Galactosidase Staining Kit according to the manufacturer's instructions. Briefly, cultured cells were treated with cisplatin as described above. After treatment, the cells were washed twice with PBS and fixed with a 3.5% paraformaldehyde solution for 15 minutes at room temperature. The cells were washed every 5 minutes 3 times in PBS, and the SA-gal stain solution (pH 6.0) was added and incubated for 16 hours at 37°C. The percentage of positively stained cells was determined by counting 3 random fields of at least 100 cells each. Images of representative fields were captured under a 200× magnification.

### Immunofluorescence

The cells were plated in six-well cell culture plates (10,000 cells per well) in which slides were previously placed for immunostaining. Twenty-four hours after plating, the cells were treated with the indicated concentration of cisplatin for 24 hours; subsequently, the cells were washed twice with PBS and maintained for 5–6 days in fresh medium containing 10% FBS until analysis. The cells were fixed in 75% ethanol for 30 minutes, permeabilized in 3% Triton X-100–PBS for 5 minutes, and blocked in 5% normal goat serum in PBS at 37°C for 1 hour. The cells were incubated with HP1-γ polyclonal antibody overnight at 4°C. After washing, the cells were incubated with the goat anti-rabbit immunoglobulin G (IgG) FITC-labeled secondary antibody for 30 minutes at room temperature. The nuclei were stained with 2.5 µg/ml DAPI solution. The slides were observed using a laser confocal microscope under a 600× magnification.

### Cell cycle profiling

For cell cycle profiling, the cells were fixed with 75% ice-cold ethanol and stained with propidium iodide (50 µg/mL) before fluorescence-activated cell sorting analysis (FACS).

### Cell proliferation analysis by CFSE

Carboxyfluorescein succinimidyl ester (CFSE) is a green fluorescent dye that is distributed equally among daughter cells with each cell division. For labeling, cells were incubated with 3 µM CFSE in serum-free DMEM for 15 minutes at 37°C. Excess dye was removed by two rinses in fresh, complete medium. The CFSE-labeled cells were plated in 6-well flat-bottom plates and cultured in fresh, complete medium with or without cisplatin for the indicated time. The cells were harvested and examined by flow cytometry. The data were analyzed using the ModFit software (Becton Dickinson). The cell proliferation model calculated the proliferation index, which is the ratio of the total number of cells analyzed to the calculated number of parent cells required to produce the observed number of cells.

### Protein preparation for 2-DE

NG108-15 cells were grown in 75 mm tissue culture flasks until ∼30–40% confluent. The cells were treated with the indicated concentration of cisplatin for 24 hours and maintained in fresh complete DMEM for 6 days to induce senescence. Subsequently, the senescent cells were harvested by treatment with 0.25% trypsin and 0.02% EDTA. Both the control cells and senescent cells were rinsed 3 times in cold PBS. The cell pellets (1×10^7^ cells each) were resuspended in 500 mL sample buffer or cell lysis solution containing 8 M urea, 4% CHAPS, 65 mM DTT, 40 mM Tris-HCl, 4 mM EDTA, 0.2% Biolyte ampholytes, Complete protease inhibitor cocktail, and 20 U benzoase nuclease. Then, the cell pellets were freeze-thawed 3 times in liquid nitrogen, sheared by aspiration five times using a 27-gauge needle to remove DNA, and centrifuged at 12,000 g for 45 minutes at 4°C. The supernatants were purified and concentrated using the Ready-Prep Cleanup kit. The soluble proteins were recovered and stored at −80°C until use. Protein concentration was determined using the Bio-Rad RC DC Protein Assay.

### 2-DE

2-DE was performed as previously described [13] using the PROTEAN IEF and PROTEAN Plus Dodeca cell systems (BioRad). Total protein (300 µg/250 µL) pre-mixed with rehydration buffer was run in an IEF system using a precast 11 cm IPG strip with a pI range of 3–10 (BioRad). Passive rehydration was performed for 12 hours to introduce the protein samples onto the IEF strip. IEF was performed as follows: slow 250 V for 30 minutes, rapid 1,000 V for 30 minutes, linear 8,000 V for 4 hours, and rapid 8,000 V for 5 hours. Each run required approximately 24 hours for completion. The total Vh ranged from 60,000–69,000. Following IEF, the IPG strips were equilibrated in equilibration buffer I (6 M urea, 2% SDS, 0.375 M Tris-HCl, pH 8.8, 20% glycerol, and 2% DTT) for 15 minutes, then equilibrated in equilibration buffer II (DTT was replaced with 2.5% IAA) for 15 minutes. The continuous 10% linear gradient SDS-PAGE gels onto which the strips were loaded were electrophoresed at a constant voltage of 120 V for 2–3 hours until the bromophenol blue reached the bottoms of the gels. The gels were stained with “blue silver”, a very sensitive colloidal Coomassie G-250 staining dye compatible with MS analysis.

### Gel scanning and image analysis

Images of gels stained with “blue silver” were obtained using a BioRad Densitometer GS 710 at 400 dpi resolution and analyzed using PDQuest software Version 7.3 (BioRad). The gel patterns of proteins from control and senescent cells run simultaneously were automatically compared using the match set method. The relative stain intensity ratio for each gel spot in the individual protein profiles was determined using the following procedure. The staining was quantified using the following formula: quantity (Q) = area×OD. The total density in a gel image was used to normalize each spot volume in the gel image to minimize the effect of experimental factors on the spot volume. Data are expressed such that a 1.5-fold quantity change in each spot from normal cells is compared to the same spot in senescent cells. The process was repeated 3 times. A reproducible change in protein expression was defined as being greater than a 1.5-fold difference between normal and senescent cells in 3 independent gels.

### In-gel trypsin digestion

“Blue silver”-stained spots were excised, cut into 1 mm cubes, placed into 96-well microtiter plates, and covered with 50 mL HPLC water. Ammonium bicarbonate, DTT, and IAA solutions were prepared fresh for each experiment. The gel pieces were destained, reduced in DTT, alkylated in IAA, and digested overnight with sequencing-grade trypsin in the MassPrep Robotic Sample Preparation Station (Bruker Daltonics) according to the manufacturer's recommended protocols.

### MALDI-TOF MS analysis

MALDI-TOF MS was performed using an autoflex MALDI reflectron TOF mass spectrometer (Bruker Daltonics) as describe previously [14]. Each sample (0.5 mL, representing ∼5% of the total digest volume) was mixed with 0.5 mL of the matrix (CHCA, 10 mg/mL in 50% ACN/0.1% TFA), spotted onto the MS plate, and air dried. The samples were analyzed by MALDI-TOF MS operating in the delayed extraction/reflectron/positive ion detection mode with an accelerating voltage of 15 kV and a 499 ns delay. A nitrogen laser (337 nm) was used to irradiate the sample, and a mass spectrum was acquired in the mass range 800–3,000 Da. The instrument was externally calibrated using five peptide standards (MH1: Angiotensin II, 1046.5420 Da; Angiotensin I, 1296.6853 Da; Substance P, 1347.7361 Da; Bombesin, 1619.823 Da; and ACTH clip 18–39, 2465.199 Da) from Bruker or internally calibrated with autodigest peaks of trypsin (MH1: 2163.057 Da, 2273.160 Da). ACTH was also used as an internal lock mass at m/z 2465.199 when added to the matrix at 100 fmol/mL.

### Protein identification

The proteins were identified by PMF using the MASCOT search engine (http://www.matrixscience.com/, MatrixScience Ltd, UK) against the NCBI non-redundant protein database (http://www.ncbi.nlm.nih.gov/). The mass tolerance was 80 ppm, and the errors in peptide masses ranged from 0.01–0.1%. One missed tryptic cleavage site per peptide was allowed during the search. Proteins matching more than four peptides and with a MASCOT score higher than 66 were considered significant (p<0.05). In MASCOT searching, the carboamidomethylation of cysteine was selected as the static modification and oxidation of variable methionine as the differential modification.

### Western blot analysis

Preparation of protein samples and Western blot analyses were carried out as described previously [15]. The immunoblotting antibodies used were the following: GRP78 (1∶500), P53 (1∶500), P21 (1∶500), p-CDC2 (1∶500), CDC2 (1∶500), and β-actin (1∶1,000). After washing in PBS, the membranes were incubated with a secondary antibody at a dilution of 1∶1000. The proteins were visualized using the BCIP/NBT immunoblotting detection system.

### Animal treatment protocol

Female athymic Balb/c nu/nu mice (5–8 weeks old; obtained from the Animal Experimental Center of Slaccas, Shanghai, China) were subcutaneously inoculated in the left flank with 2×10^6^ NG108-15 cells mixed with an equal volume of saline. After 8 days, the mice were randomly sorted into 2 groups (n = 5 for each group) reflecting the different treatment regimens. Group 1 received only a saline injection, and group 2 was injected twice with cisplatin at a dose of 10 mg/kg, with a 3 days interval between injections. The mice were monitored twice a week by inspection and palpation. 7 days after the first injection, the subcutaneous tumors were removed, freshly frozen in Tissue-Tek cryopreservation medium, cryostat sectioned, and stored at −20°C for SA-β-galactosidase staining and immunohistochemical analysis.

### Immunohistochemical analysis

The immunohistochemical analysis was carried out as described previously [16]. The negative control slides were processed similarly but with the omission of the primary antibody.

### Transient siRNA transfection

The annealed siRNAs were synthesized by RiboBio Co, Ltd. (Guangzhou, China). The BiP/GRP78-specific siRNA (sense sequence, 5′-GAGUGACAGCUGAAGACAAdTdT-3′; antisense sequence, 5′-GCCAGCGCACCTdTd-3′) was used to knock down the expression of BiP/GRP78. The NG108-15 cells were plated onto six-well cell culture plates at a 30% cell density. After 24 hours, the cells were transfected with GRP78 siRNA or control siRNA using Lipofectamine 2000 (Invitrogen) according to the manufacturer's instructions. Briefly, for each well in a six-well plate, 5 µl of Lipofectamine 2000 was diluted in 0.25 ml of serum-free DMEM. This mixture was carefully added to a solution containing 50 nmol of siRNA in 0.25 ml of serum-free DMEM. The solution was incubated for 20 minutes at room temperature and then gently overlaid onto cells that were 40–50% confluent in 1.5 ml of serum-free DMEM. Four-to-six hours after transfection, the cells were cultured in fresh complete medium for 48 hours before exposure to cisplatin.

### Determination of relative [Ca^2+^] concentration

Fluo3-AM (Invitrogen) exhibits a large increase in fluorescence intensity upon binding Ca^2+^ following excitation at 488 nm using an argon-ion laser. Briefly, the cells were plated onto 35 mm glass bottom dishes (MATTEK, USA). 12 hours after treatment, the control and treated cells were washed 3 times with D'-Hank's, incubated with serum-free DMEM containing 4 µM Fluo3-AM and 0.05% Pluronic-F127 for 45 minutes at 37°C while being protected from the light. Next, the cells were washed twice with Hank's containing 0.2% BSA and subsequently washed with Hank's to remove the residual Fluo3-AM.The cells were observed using laser confocal microscope with a 488 nm excitation wavelength and 505–530 nm absorption wavelength (60× objective lens, 1024×1024 resolution) at 37°C. Ten fluorescent images were collected at 10 second intervals. The mean Ca^2+^ fluorescence intensity was analyzed using the FLUOVIEW software. 3 independent experiments were performed, and 10 cells were monitored for each experiment.

### Statistical analysis

The differences in expression between normal and senescent cells were determined by calculating p-values using Student's t-test. Statistical significance between groups was determined by ANOVA analysis and defined as P<0.05(*).

## Results

### Cisplatin induces senescence in NG108-15 cells

**Figure 1:**
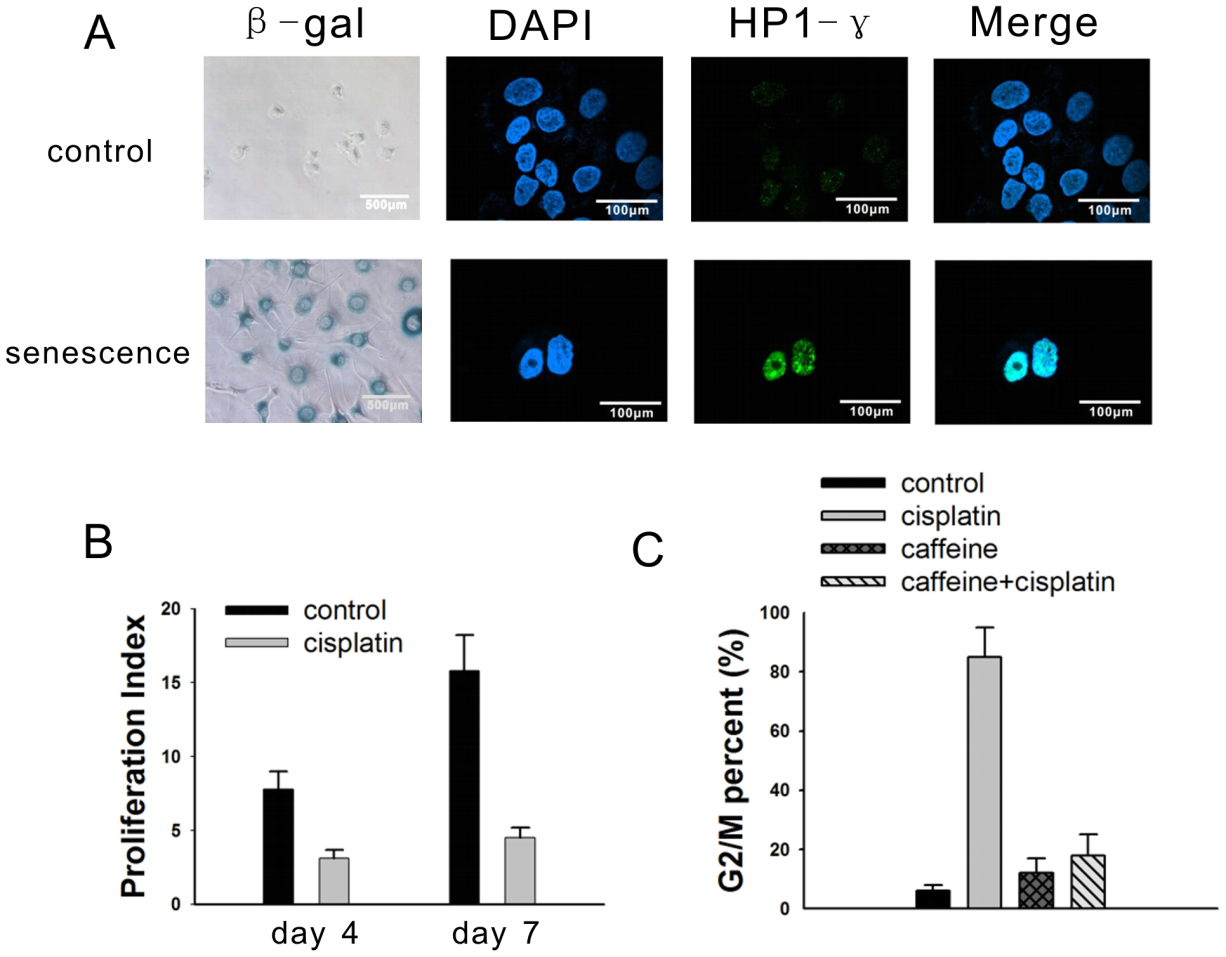
Cisplatin induces a senescent phenotype in NG108-15 cells. (A) Untreated cells and cells treated with 5 µg/ml cisplatin were stained with β-gal at pH 6.0 (200×, light microscopy), DAPI (600×, confocal microscopy), and immunofluorescent HP1-γ protein (600×, confocal microscopy). The DAPI and HP1-γ stains were merged. (B) The cells were labeled by CFSE to determine the proliferation index on day 4 and day 7 following cisplatin treatment. The proliferation index of the untreated cells on 0 hour was 1. (C) 7 days after treatment with 5 µg/ml cisplatin, 5 mmol/L caffeine, or a combination of cisplatin and caffeine, the NG108-15 cells were harvested for FACS analysis. The results are the mean of 3 independent experiments.

To determine whether cisplatin could induce a senescence-like phenotype, the NG108-15 cells were exposed to 2.5 µg/ml, 5 µg/ml or 10 µg/ml cisplatin for 24 hours. After 6 days of recovery in complete medium, the NG108-15 cells exhibited the maximum proportion of senescence phenotype when treated with 5 µg/ml cisplatin (Figure S1A). Nearly 90% of the tumor cells showed an enlarged, flattened morphology, with increased cytoplasmic granularity and positive staining for SA-gal (Figure 1A and Figure S1A), as well as expression of the senescence-associated heterochromatic foci (SAHF) marker heterochromatin protein 1-γ (HP1-γ) (Figure 1A and Figure S1B). These senescent cells did not divide further, as confirmed by the CFSE proliferative index (PI). The PI values were 3.1±0.8 and 4.5±0.7 on day 4 and day 7 in the cisplatin-treated cells, respectively, while the PI values were 7.8±1.2 and 15.8±2.4 in the control cells on day 4 and day 7, respectively. (Figure 1B and Figure S2). According to previous report [10], caffeine could relieve the ataxia telangiectasia mutated (ATM)-related G2/M arrest. In our data, the NG108-15 cells were arrested in G2/M phase on day 7 after cisplatin treatment, and this effect was mediated by the ATM gene, which was verified by the ability of caffeine pretreatment (5 mmol/L) to relieve the cisplatin-induced G2/M cell cycle arrest (Figure 1C and Figure S3).

### Comparative proteomic analysis of normal and senescent NG108-15 cells

**Figure 2:**
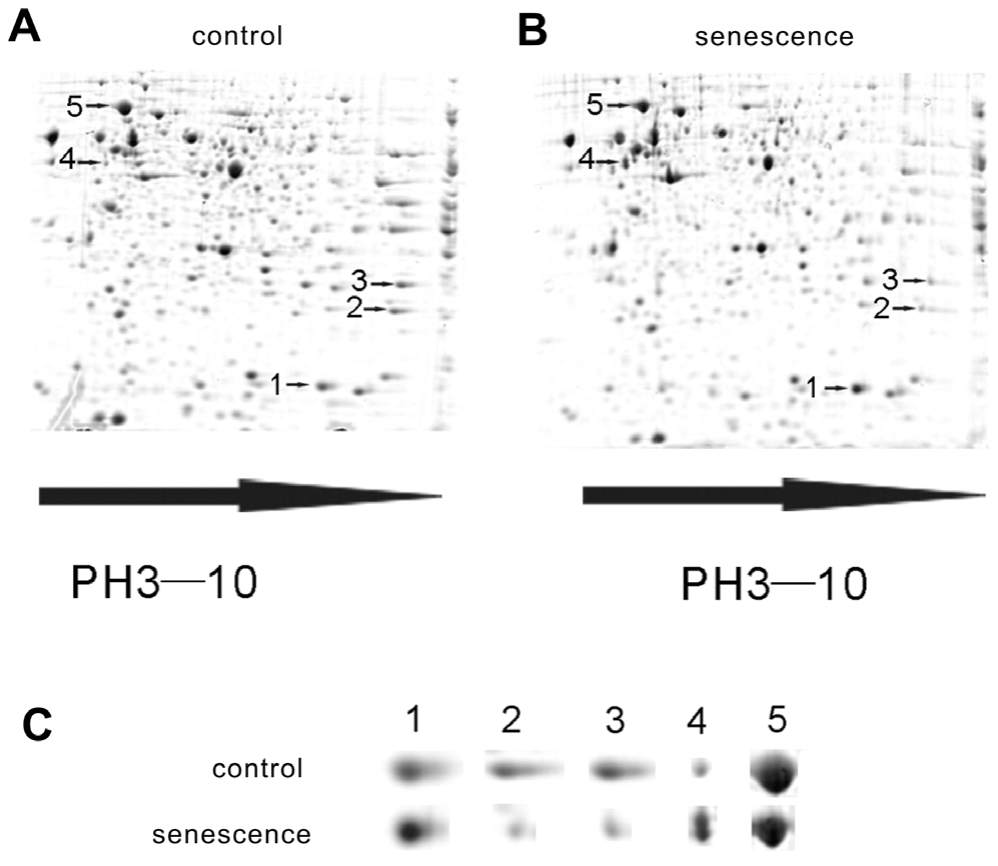
Proteomic analysis of normal and senescent NG108-15 cells. (A) and (B) Representative 2-DE gel patterns of untreated NG108-15 cells and cells treated with 5 µg/ml cisplatin. The 2-DE patterns of intracellular proteins were visualized using the “blue silver” dye. Each gel, which contained 260 mg of protein, showed more than 400 spots. 3 protein spots (2, 3, and 5, designated by arrow heads) were expressed at higher levels in the untreated cells, and two protein spots (1 and 4, designated by arrow heads) were expressed at higher levels in the cisplatin-treated NG108-15 cells. (C) The 2-DE patterns of the untreated cells (top) and cisplatin-treated NG108-15 cells (bottom). The spot numbers in (C) correspond to those in (A) and (B).

To find the genes that may play key roles in cisplatin induced-senescence, the differences in the protein profiles of normal and senescent cells were examined using 2-dimensional electrophoresis (2-DE). First, three 2-DE gels of normal NG108-15 cells and three 2-DE gels of senescent cells were compared. The “blue silver”-stained 2-DE gels of the normal and senescent cell proteomes are shown in Figure 2A and Figure 2B, respectively. We detected 517±25 and 474±21 protein spots in the normal and senescent cells, respectively. One of the gels from the normal cells was selected as a reference gel, and 65% of its protein spots could be matched to the gels from the senescent cells. The correlation coefficient between the normal and senescent cells was approximately 76%. The quantitative differences between the spots from the normal and senescent cells were compared and analyzed using the PDQuest software. As shown in Figure 2C, there were 5 protein spots with different expression levels in the normal and senescent cells. Two protein spots appeared to be more intense and 3 spots appeared to be less intense in the senescent cells, compared to the control cells.

**Figure 3:**
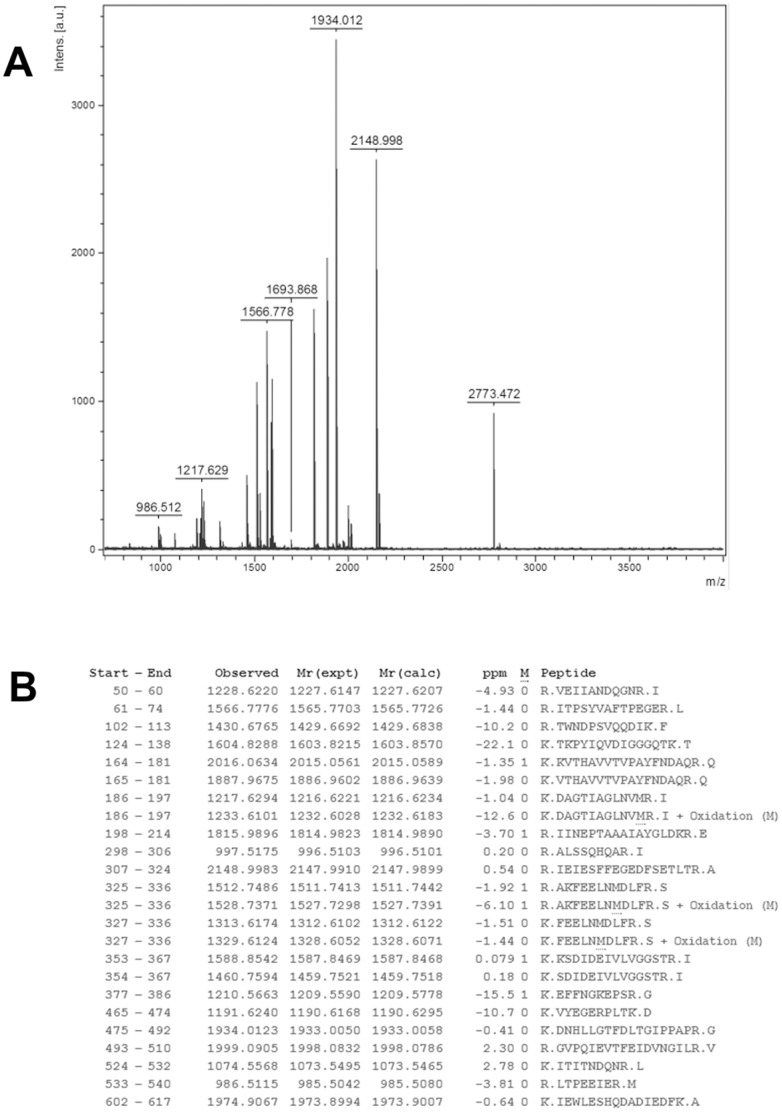
MALDI-TOF mass spectrum analysis of GRP78. (A) MALDI-TOF mass spectrum obtained from spot 5 after trypsin digestion. The mass spectrum of GRP78, with a precursor ion *m/z* of 1934.012, is indicated. (B) Peptide sequences from GRP78 matched with the peaks obtained from the mass spectrum. No matches were found for the peaks at 1211.5525, 1464.7470, 1476.5912, 1580.8024, 1592.7943, 1693.8678, 1916.9787, 2163.0147, 2773.4722, and 2807.2904. The data are representative of the results from 3 independent experiments.

Next, the selected protein spots from the “blue silver”-stained gels were excised and subjected to in-gel tryptic digestion. The extracted peptides were analyzed by MALDI-TOF MS to generate PMFs. Figure 3A showed a MALDI-TOF mass spectrum of peptides derived from protein spot 5. A MASCOT search using the PMF data indicated that 24 peptides matched the peptides from GRP78, which represents a sequence coverage of 37% (Figure 3B). The experimental masses of thirteen GRP78 peptides exactly matched their theoretical masses, without any deviation. The score given by MASCOT was as high as 239, indicating significant confidence (p<0.05) in the identification. A total of 5 differentially expressed protein spots were successfully identified using the PMF data from the MALDI-TOF analysis. The summary scores, protein coverage and quantity of the spots derived from the normal and senescent cells are presented in Table 1. Both the PPIA and VIM proteins were significantly up-regulated in the senescent cells compared to the control cells. In contrast, PRX1, GSTM1 and GRP78 were notably down-regulated in the senescent cells compared to the control cells.

**Table 1:** List of identification results for the proteins that were differentially expressed between the normal NG108-15 cells and the senescent NG108-15 cells.

Spot	Protein Description^a^	Accession no.^b^	Theoretical Mr(kDa)/pI	Sequence Coverage^c^	Summary Score^d^	Protein Level^e^	*p*-value^f^	Normal(mean±SD)^g^	Senescence(mean±SD)^g^
1	peptidylprolyl isomerase A	NP_032933	18.131/7.74	0.31	83	↑	<0.01	31342±523	51245±662
2	peroxiredoxin 1	NP_035164	22.390/8.26	0.63	184	↓	<0.01	16783±726	5722±357
3	glutathione S-transferase, mu 1	NP_034488	26.067/7.71	0.71	187	↓	<0.01	19886±548	9937±469
4	vimentin	CAA69019	51.590/4.96	0.57	267	↑	<0.01	5170±536	29865±418
5	glucose-regulated protein 78	NP_037215	72.473/5.07	0.37	239	↓	<0.01	82853±758	42305±477

a . According to SWISS-PROT DE line.

b . SWISS-PROT accession number.

c . Ratio of matched peptides with total PMF peptides.

d . Percent of identified sequence to the complete sequence of the known protein.

e . The increased or decreased expression protein levels in the Senescent NG108-15 cells as compared to the Normal NG108-15 cells and (±) indicates identical spot intensity in the two gels.

f . The *p*-value was statistically analyzed by student *t*-test analysis between Normal NG108-15 cells and NG108-15 cells.

g . The quantity (mean ± SE) of each protein spot was calculated using 2-DE gels.

### Verification of the differentially expressed proteins

**Figure 4:**
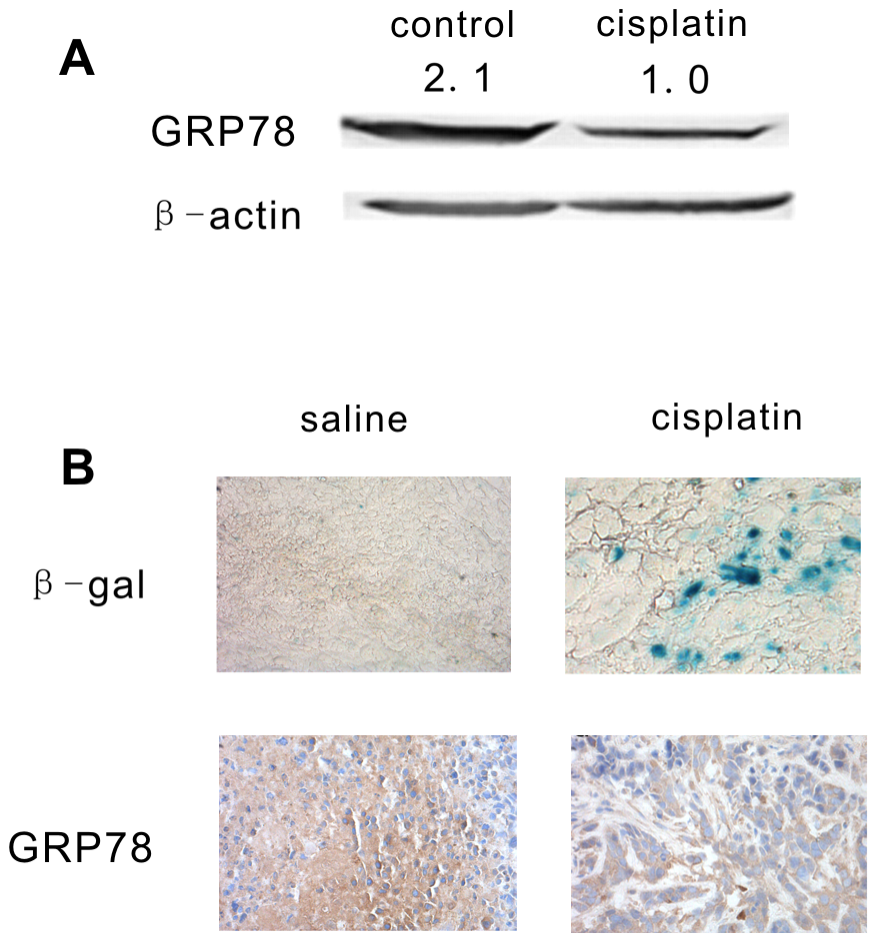
GRP78 expression *in vitro* and *in vivo*. (A). NG108-15 cells were treated with 5 µg/ml cisplatin, and the GRP78 protein level was examined and quantified by western blot analysis on day 7 following cisplatin treatment. (B). Female athymic Balb/c nu/nu mice were subcutaneously inoculated with 2×10^6^ NG108-15 cells. After 8 days, the mice were randomly sorted into 2 groups, which received saline injection or cisplatin injection. The subcutaneous tumors sections were stained with SA-β-galactosidase (Upper Row) and GRP78 (Lower Row). The data are representative of the results from 3 independent experiments.

Of the identified candidate proteins, GRP78 was the most abundant and well-characterized glucose-regulated protein, and this protein has been shown to be responsible for the growth and apoptosis capacity of many malignant tumors. Therefore, GRP78 was further validated *in vitro* and *in vivo*. As shown in Figure 4A, cisplatin treatment resulted in decreased GRP78 protein levels by more than 50% in the senescent NG108-15 cells, which was consistent with that of the proteomic analysis. *In vivo*, the NG108-15 tumor-bearing nude mice were treated with saline or cisplatin for 7–10 days, and the subcutaneous tumor sections were stained for senescence-associated β-galactosidase and GRP78. The cisplatin-treated tumor sections showed positive staining for SA-β-gal (Figure 4B, Upper Row), and GRP78 staining in the cisplatin-treated tumor sections was significantly weaker than that of the saline- treated tumor sections (Figure 4B, Lower Row).

### Up-regulation of GRP78 expression can protect NG108-15 cells from cisplatin-induced senescence, which was accompanied by obvious suppression of P53 expression

**Figure 5:**
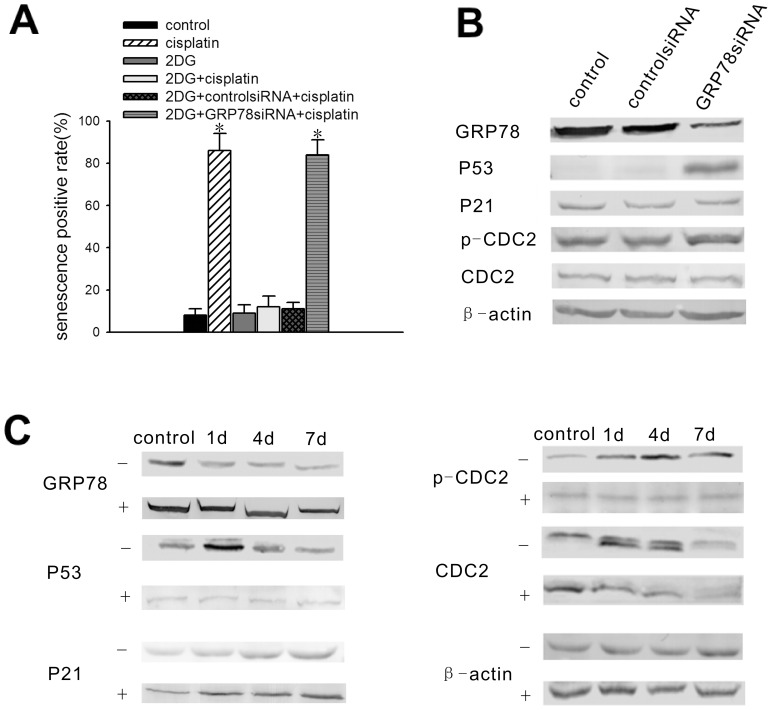
The up-regulation of GRP78 expression results in resistance of the NG108-15 cells to cisplatin-induced senescence, requiring the suppression of P53 expression. NG108-15 cells were treated with 2DG at a final concentration of 10 nmol/ml for 1 day prior to cisplatin treatment or siRNA transfection. (A) Senescence-positive staining rate of NG108-15 cells. Before 5 µg/ml cisplatin treatment, the cells were induced with or without 2DG and were transfected with GRP78 siRNA or control siRNA. The cells were stained with β-gal at pH 6.0 on day 7 following cisplatin treatment. The senescence rates are presented as the mean ± standard error (n = 3). (B) Expression of the ATM pathway genes in NG108-15 cells after GRP78 knockdown. GRP78, P53, P21, p-CDC2, and CDC2 protein levels were examined by western blot analysis 2 days after GRP78 siRNA transfection or control siRNA transfection. (C) GRP78, P53, P21, p-CDC2 and CDC2 protein levels were examined by western blot analysis on day 1, day 4, and day 7 following 5 µg/ml cisplatin treatment in the presence or absence of 2DG induction. −: without 2DG treatment, +: with 2DG treatment. The data are representative of the results from 3 independent experiments. *Significantly different from the control value (P<0.05).

To determine the role that GRP78 plays in cisplatin induced senescence, 2DG was used to increase GRP78 expression; this compound is a non-metabolizable glucose analogue that was previously shown to up-regulate GRP78 expression. In our experiment, 2DG was added to the medium of the NG108-15 cells at a final concentration of 10 nmol/ml for 24 hours prior to treatment with 5 µg/ml cisplatin. As shown in Figure 5A, the percentage of SA-gal-positive, 2DG-treated NG108-15 cells after 7 days cisplatin treatment was nearly 20 percent of that for the cisplatin-treated cells without 2DG treatment. Compared to the enlarged, flattened morphology typical of senescent cells, the 2DG-treated NG108-15 cells showed slender, branch-like morphology following cisplatin treatment (Figure S4). To confirm the specific anti-senescence effect of GRP78, GRP78 expression was knocked down using GRP78 siRNA in NG108-15 cells, which were previously treated with 2DG for 24 hours (Figure S5). Then, the NG108-15 cells were incubated with fresh complete medium for 2 days prior to cisplatin treatment and were stained with SA-gal 7 days after cisplatin treatment. The percentage of SA-gal positive-cells returned to a level similar to that of the cisplatin-treated cells (Figure 5A and Figure S4). It is plausible that the 2DG-induced GRP78 overexpression can protect NG108-15 cells from cisplatin-induced senescence.

To explore the anti-senescence mechanisms of GRP78, NG108-15 cells were transfected with GRP78 siRNA or control siRNA. The results showed that GRP78 protein levels decreased significantly after transfection of GRP78 siRNA into NG108-15 cells. Additionally, P53 levels increased significantly when GRP78 was depleted from the cells. Nonetheless, there were no changes in the protein levels of P21, p-CDC2 and CDC2 (Figure 5B). Furthermore, P53, P21, p-CDC2 and CDC2 levels were determined using western blot analysis on day 1, day 4, and day 7 following cisplatin treatment, with or without 2DG treatment. In the non-2DG-treated cells, GRP78 expression decreased from day 1 to day 7, P53 expression increased significantly on day 1 and decreased to baseline on day 4 and day 7, and P21 expression increased significantly on day 1 and maintained a high level until 7 days following cisplatin treatment. Total CDC2 suddenly decreased on day 7, while p-CDC2 increased significantly after exposure to cisplatin. In the 2DG-treated cells, GRP78 expression increased significantly and was maintained at a high level after cisplatin treatment. However, there were no changes in the protein levels of P53 and p-CDC2 during cisplatin exposure. The changes in the protein levels of CDC2 and P21 were consistent with those of the non-treated cells (Figure 5C). Above all, it appeared that P53 was negatively associated with GRP78, which may be partially responsible for its anti-cisplatin induced senescence.

### 2DG-induced GRP78 overexpression could prevent cisplatin induced-senescence through the inhibition of Ca^2+^ release from ER stores

**Figure 6:**
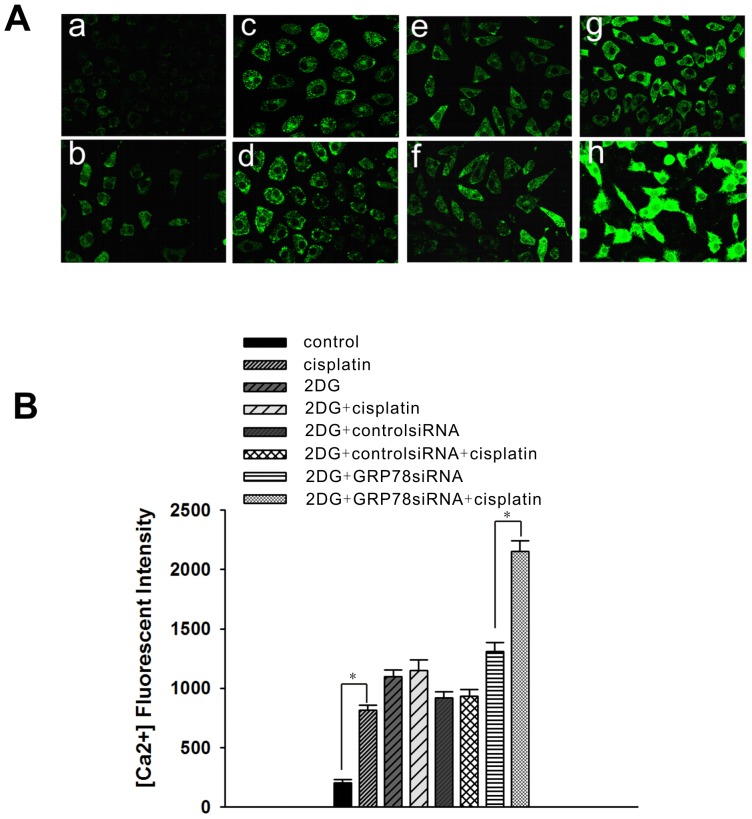
Relative cytoplasmic Ca^2+^ concentration in NG108-15 cells. NG108-15 cells were treated with 5 µg/ml cisplatin for 12 hours. A final concentration of 10 nmol/ml 2DG was added 1 day prior to cisplatin treatment. After 2DG treatment for 24 hours, the NG108-15 cells were transfected with GRP78 siRNA or control siRNA, and the cells were cultured in fresh complete medium for 48 hours before cisplatin treatment. (A) The Ca^2+^ content of the cells was stained with Fluo3-AM (600×). a: untreated NG108-15 cells; b: cisplatin-treated NG108-15 cells; c: 2DG-induced NG108-15 cells; d: 2DG + cisplatin-treated NG108-15 cells; e: 2DG + control siRNA-treated NG108-15 cells; f: 2DG + control siRNA + cisplatin-treated NG108-15 cells; g: 2DG + GRP78 siRNA-treated NG108-15 cells; h: 2DG + GRP78 siRNA + cisplatin-treated NG108-15 cells. (B) Ca^2+^ content of the cells was analyzed using the FLUOVIEW software. The data are shown as the mean ± SE of 3 independent experiments. *Significantly different (P<0.05).

GRP78/BiP is a major endoplasmic reticulum chaperone with Ca^2+^-binding properties that is involved in ER calcium homeostasis. Therefore, we examined the changes in the relative cellular Ca^2+^ concentration to see whether it was relevant to the anti-senescence effect of GRP78. In NG108-15 cells, the cellular Ca^2+^ concentration was elevated by approximately 4-fold after exposure to 5 µg/ml cisplatin for 12 hours. After 2DG induction for 24 hours, the cellular Ca^2+^ concentration was significantly elevated, but did not increase further after cisplatin treatment for 12 hours. Nonetheless, when GRP78 expression was suppressed by GRP78 siRNA following 2DG treatment, the cellular Ca^2+^ concentration was significantly elevated at 12 hours after cisplatin treatment (Figure 6A and Figure 6B). This suggests that GRP78 may prevent the cisplatin induced-senescence through the inhibition of Ca^2+^ release from the ER stores.

## Discussion

In this study, we found that cisplatin could induce senescence in NG108-15 cells. According to 2-dimension electrophoresis, we found that there were 5 differentially expressed proteins between the normal and senescent NG108-15 cells: PPIA, PRX1, GSTM1, VIM, and GRP78. Furthermore, this study verified that GRP78 expression was negatively associated with cisplatin-induced senescence *in vitro* and *in vivo*. Knockdown of GRP78 expression rescued the senescence sensitivity of the NG108-15 cells to cisplatin. The ATM pathway genes *P53*, *P21*, *CDC2* and ER calcium homeostasis were involved in the cisplatin-induced senescence.

PPIA is a member of the peptidylprolyl cis-trans isomerase (PPIAse) family. PRX1 is a member of the peroxiredoxin family of antioxidant enzymes, which reduce hydrogen peroxide and alkyl hydroperoxides. It was identified that PPIA and PRX1 expression were both increased in the temporal cortex of aged rats [17]. Nonetheless, the roles of PPIA and PRX1 in senescence have not yet been explored. Our data showed that PPIA significantly increased and PRX1 significantly decreased in the senescent NG108-15 cells treated with cisplatin. This suggested that PPIA and PRX1 may play roles in cisplatin-induced senescence.

GSTM1 is a cytoplasmic glutathione S-transferase that belongs to the mu class. Null mutations in this mu class gene have been associated with an increased rate of a number of cancers, likely due to an increased susceptibility to environmental toxins and carcinogens [18]. There are few reports about the relationship between senescence and GSTM1. The results showed that GSTM1 expression decreased significantly in the senescent NG108-15 cells.

Vimentin is a type III intermediate filament (IF) protein that is expressed in mesenchymal cells. All IF proteins are expressed in a highly developmentally regulated manner, with vimentin being the major cytoskeletal component of mesenchymal cells. Because of this, vimentin is often used as a marker of mesenchymal-derived cells or cells undergoing the epithelial-to-mesenchymal transition (EMT) during both normal development and metastatic progression. One characteristic feature of senescent fibroblasts is their flat, enlarged, and heterogeneous cell shapes. It has been suggested that senescent fibroblasts overproduce vimentin, and the overproduced vimentin filaments bring about the senescent cell morphology [19]. Our data also showed that Vimentin was significantly increased in the senescent NG108-15 cells.

GRP78, the most abundant and well-characterized glucose-regulated protein, is a major stress-inducible chaperone localized in the ER. GRP78 has been examined in human breast carcinoma, and its overexpression has been observed in most of the more aggressive, estrogen receptor-negative tumors [20]. Preliminary analysis of GRP78 in a series of primary and recurrent breast, prostate, and lung cancer samples suggests a correlation between GRP78 overexpression, recurrence, and drug resistance [21]. Moreover, GRP78 is increasingly being used as a potent target for the diagnosis and treatment of different cancers [22]. Synthetic chimeric peptides targeting GRP78 can suppress tumor growth in xenograft and isogenic mouse models of breast and prostate cancer [23]. As a calcium binding protein in the ER, GRP78 is thought to regulate the balance between cell survival and apoptosis through direct or indirect interactions with specific caspases and other upstream components of the pro-apoptotic pathways that are initiated from the ER [7], [8]. Additionally, many studies have shown that knockdown of GRP78 could increase chemotherapy sensitivity in malignant tumor cell lines [21], [24]. Nonetheless, it is still unclear whether GRP78 was associated with chemotherapy-induced ACS.

In our study, as one of the differentially expressed proteins in the normal and senescent NG108-15 cells, GRP78 was found to decrease significantly during cisplatin-induced senescence *in vitro* and *in vivo*. To clarify the role of GRP78 in cisplatin-induced senescence, GRP78 was significantly up-regulated after 2DG induction and was knocked down using GRP78 siRNA. The results showed that the up-regulation of GRP78 could confer resistance to cisplatin-induced senescence in the NG108-15 cells, which could be reversed by depletion of GRP78. It was suggested that GRP78 mediated the cisplatin-induced senescence in NG108-15 cells.

ATM pathway genes are closely associated with senescence. Therefore, we were interested in the relationship between GRP78 and the ATM pathway genes during cisplatin-induced senescence in NG108-15 cells. In cells pretreated with 2DG, P53 expression did not increase after cisplatin treatment. Meanwhile, depletion of GRP78 resulted in noticeably increased P53 levels. Many studies have shown that wild-type P53 limits cellular proliferation by inducing senescence, and this outcome is dependent on the expression level and cellular context [25], [26], [27]. An increase in P53 transcriptional activity is a molecular signature for cellular senescence [28]. The similarities associated with the suppression of the P53 pathway and the default senescence program have been attributed to certain chaperones in the Hsp70 family, including Hsp70-2 [29], Hsp72 [30], and Grp75 [31]. This suggests that suppression of P53 may contribute to the anti-senescence effect of GRP78.

CDC2 is one of the most important proteins controlling the cell cycle transition from G2 phase to M phase. Decreased CDC2 expression and increased expression of its non-active, phosphorylated (Tyr15) form often occur during G2/M phase arrest and senescence, and both of them have been established to be associated with the senescence sensitivity of tumor cells to chemotherapy [9], [10]. In our study, p-CDC2 levels did not significantly increase following cisplatin treatment in the 2DG-treated cells, which may be partially responsible for the resistance to cisplatin-induced senescence of NG108-15 cells that is caused by GRP78 up-regulation.

Cytoplasmic calcium concentrations are associated with apoptosis [32]. It was also reported that cisplatin could increase the cytoplasmic calcium concentration depending on the internal calcium store in ovarian cancer cells [33]. Nonetheless, there are few references describing the relationship between calcium and senescence. In our study, we investigated the relationship between the cytoplasmic calcium concentrations and cisplatin-induced senescence. We found that the cytoplasmic calcium concentrations were significantly elevated after cisplatin treatment, which was accompanied by senescence. Additionally, 2DG treatment could increase the cytoplasmic calcium concentration without causing senescence. This may be because the mechanism of increasing the cytoplasmic calcium concentration was different between the 2DG and cisplatin-treated cells [33]–[35]. In addition to the changes in cytoplasmic calcium, cisplatin may cause senescence through other mechanisms, such as P53 up-regulation. Moreover, the 2DG-induced GRP78 up-regulation could significantly inhibit cisplatin-induced calcium efflux from the ER into the cytoplasm, which protected the cells from cisplatin-induced senescence. Conversely, knockdown of GRP78 following 2DG induction decreased GRP78 expression to basal levels, which could restore calcium efflux from the ER to the cytoplasm with cisplatin treatment and the sensitivity of the cells to cisplatin-induced senescence. It was suggested that the GRP78-mediated changes in cytoplasmic calcium were associated with cisplatin-induced senescence.

In conclusion, our data show that the expression levels of 5 proteins were obviously changed when the NG108-15 cells were induced to undergo senescence by cisplatin treatment. As an ER protein, GRP78 confers resistance to cisplatin-induced senescence mainly through the suppression of P53 and p-CDC2. Calcium efflux from the ER to cytoplasm, which was mediated by GRP78, was also associated with the resistance to cisplatin-induced senescence. GRP78 may represent a novel target for the study of cisplatin-induced senescence in tumor cells.

## Supporting Information

Figure S1 **The effect of cisplatin-induced senescence in NG108-15 cells.** (A) The β-gal positive rate in the untreated NG108-15 cells or NG108-15 cells treated with 2.5 µg/ml, 5 µg/ml or 10 µg/ml cisplatin. The cells were allowed to recover for 6 days following cisplatin treatment. (B) The HP1-γ foci-positive rate in the untreated NG108-15 cells or in NG108-15 cells treated with 5 µg/ml cisplatin. The cells were allowed to recover for 6 days following cisplatin treatment.
(TIF)

Figure S2 **The proliferation index of the NG108-15 cells was examined by CFSE.**
(TIF)

Figure S3 **The cell cycle of the NG108-15 cells was analyzed by FACS.**
(TIF)

Figure S4 **The effect of GRP78 on cisplatin-induced senescence in NG108-15 cells.** The cells were induced with or without 2DG and were treated with GRP78 siRNA or control siRNA following cisplatin treatment. The cells were stained with β-gal at pH 6.0 on day 7 following cisplatin treatment.
(TIF)

Figure S5 **GRP78 expression in the NG108-15 cells after 2DG induction.** The cells were treated with 2DG for 24 hours. The cells were then transfected with control siRNA or GRP78 siRNA and were cultured in fresh complete medium for 48 hours prior to western blot analysis.
(TIF)
