# Supplementary figures and images for: Proteomics Analysis of Normal and Senescent NG108-15 Cells: GRP78 Plays a Negative Role in Cisplatin-Induced Senescence in the NG108-15 Cell Line
Source: PLoS One. 2014 Mar 12;9(3):e90114. doi: 10.1371/journal.pone.0090114 (PMC3951507; doi:10.1371/journal.pone.0090114)

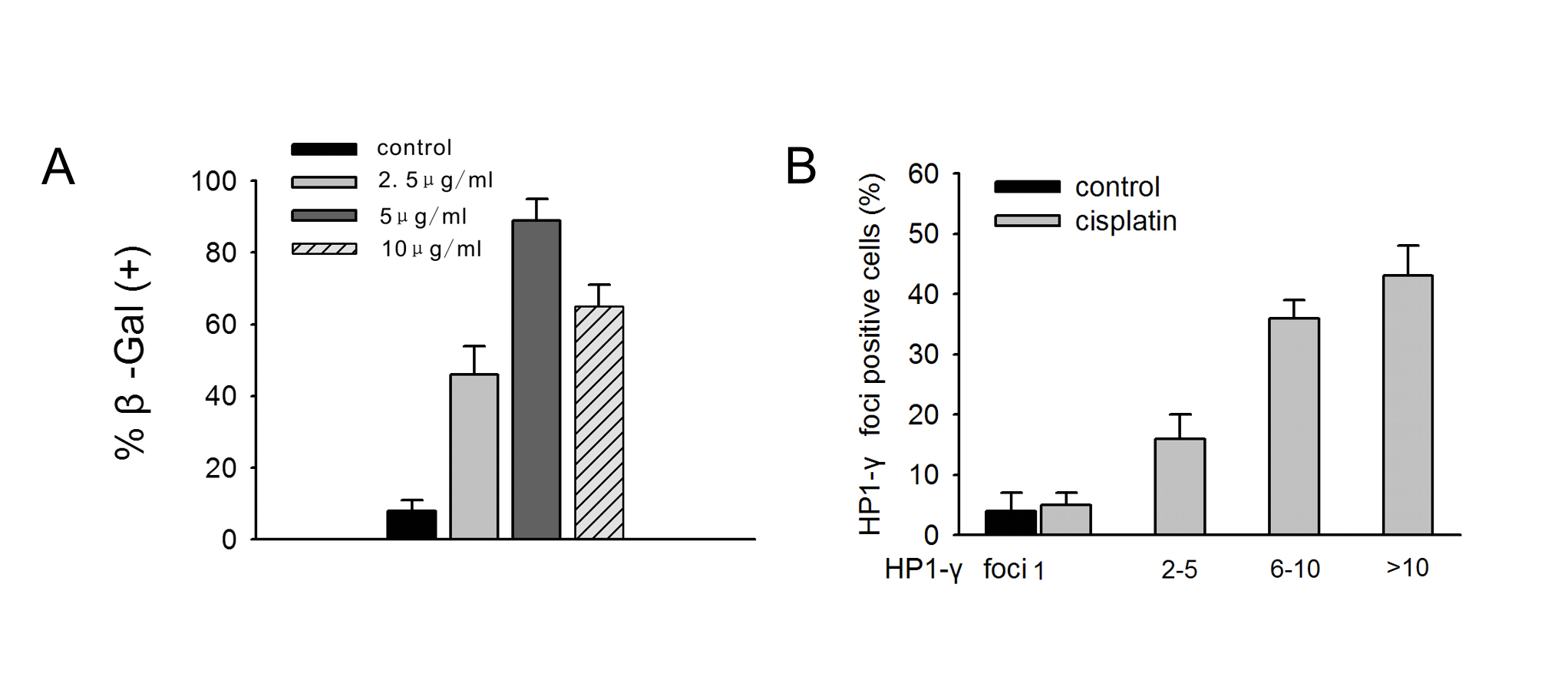

Supplement: Figure S1 — The effect of cisplatin-induced senescence in NG108-15 cells. (A) The β-gal positive rate in the untreated NG108-15 cells or NG108-15 cells treated with 2.5 µg/ml, 5 µg/ml or 10 µg/ml cisplatin. The cells were allowed to recover for 6 days following cisplatin treatment. (B) The HP1-γ foci-positive rate in the untreated NG108-15 cells or in NG108-15 cells treated with 5 µg/ml cisplatin. The cells were allowed to recover for 6 days following cisplatin treatment. (TIF) [file pone.0090114.s001.tif]

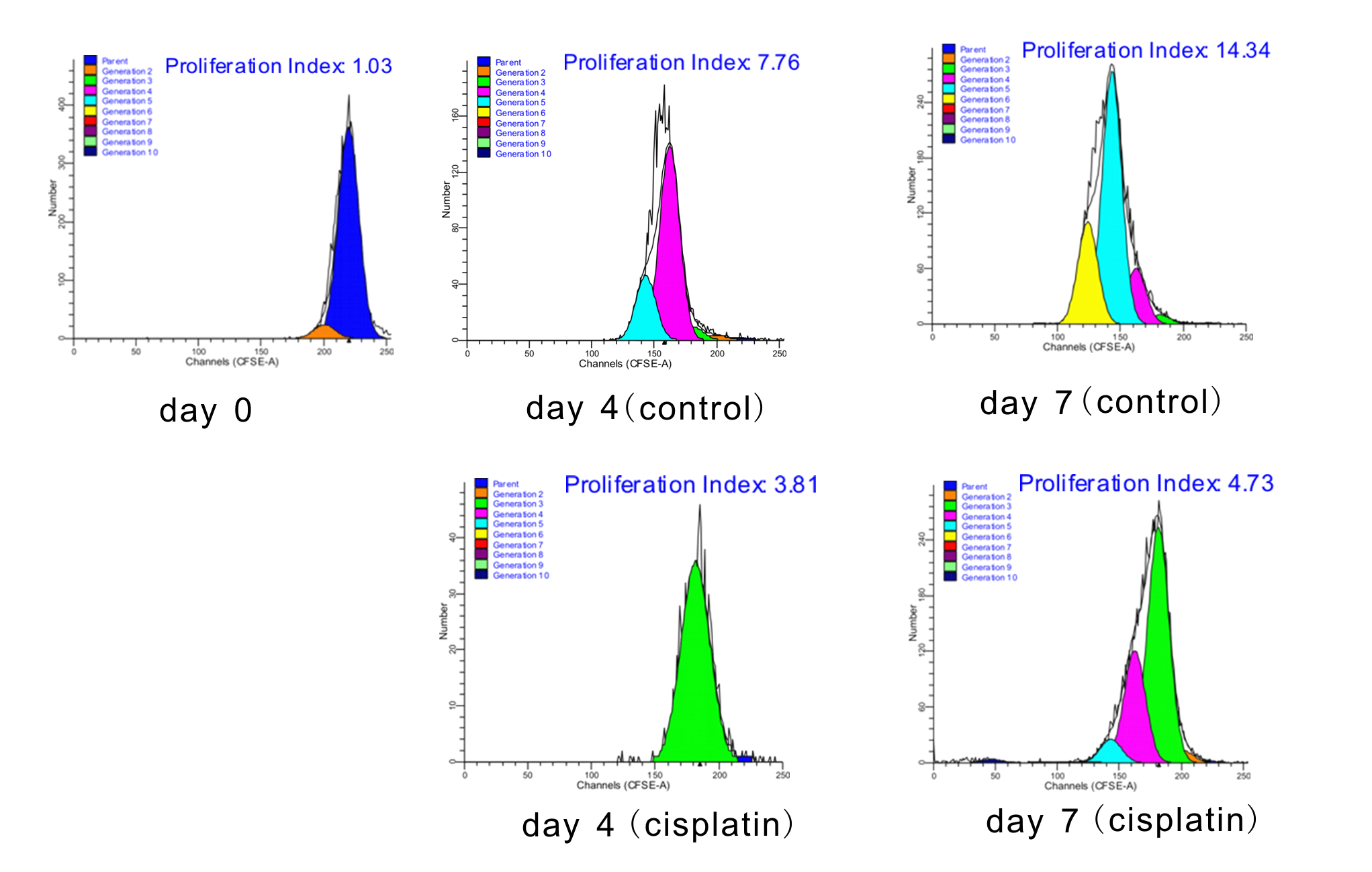

Supplement: Figure S2 — The proliferation index of the NG108-15 cells was examined by CFSE. (TIF) [file pone.0090114.s002.tif]

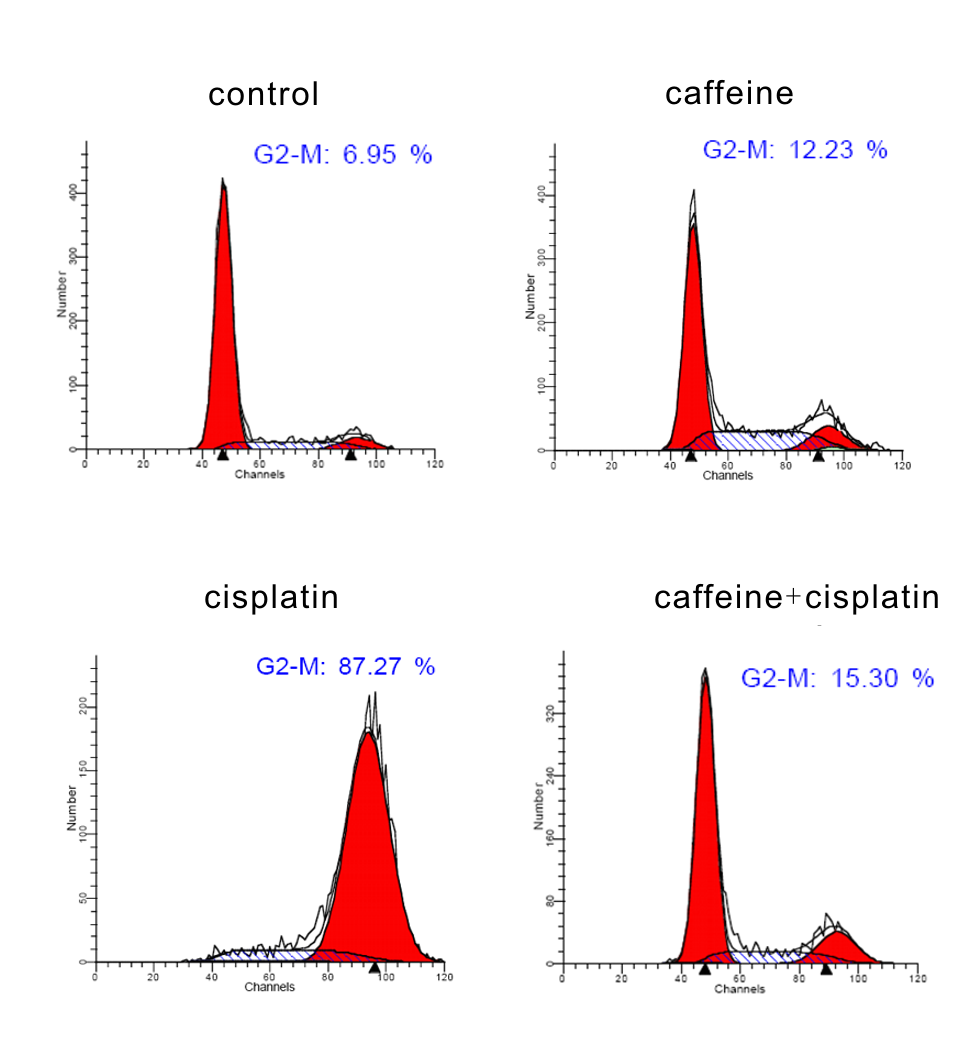

Supplement: Figure S3 — The cell cycle of the NG108-15 cells was analyzed by FACS. (TIF) [file pone.0090114.s003.tif]

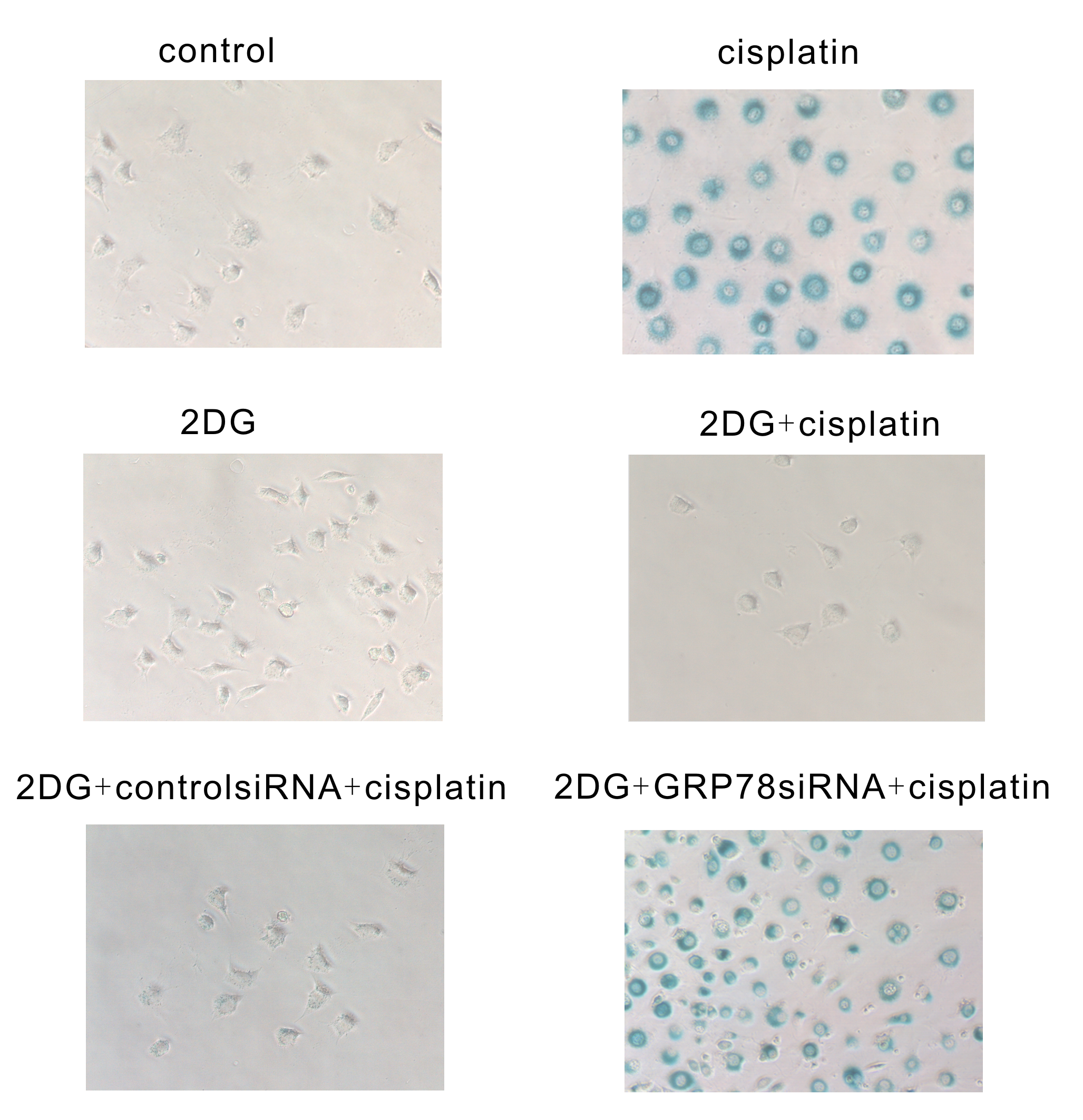

Supplement: Figure S4 — The effect of GRP78 on cisplatin-induced senescence in NG108-15 cells. The cells were induced with or without 2DG and were treated with GRP78 siRNA or control siRNA following cisplatin treatment. The cells were stained with β-gal at pH 6.0 on day 7 following cisplatin treatment. (TIF) [file pone.0090114.s004.tif]

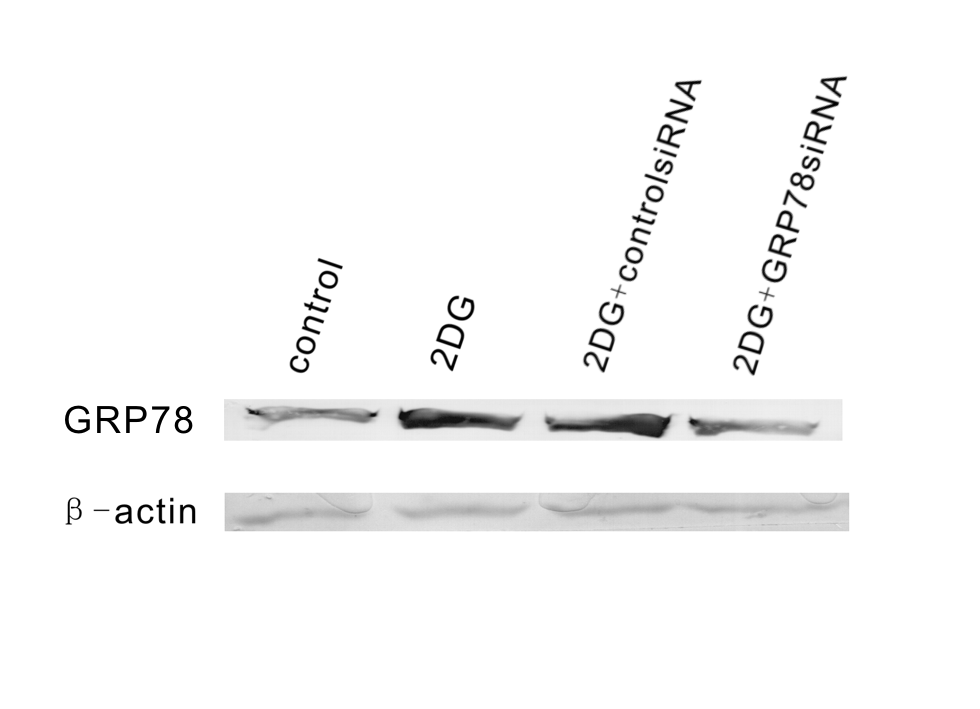

Supplement: Figure S5 — GRP78 expression in the NG108-15 cells after 2DG induction. The cells were treated with 2DG for 24 hours. The cells were then transfected with control siRNA or GRP78 siRNA and were cultured in fresh complete medium for 48 hours prior to western blot analysis. (TIF) [file pone.0090114.s005.tif]
